# Long COVID: Complications, Underlying Mechanisms, and Treatment Strategies

**Published:** 2023-05-09

**Authors:** Farigol Hakem Zadeh, Daniel R. Wilson, Devendra K. Agrawal

**Affiliations:** Department of Translational Research, College of Osteopathic Medicine of the Pacific Western University of Health Sciences, Pomona, California 91766, USA

**Keywords:** Anxiety, Brain fog, Chronic fatigue syndrome, Cognitive dysfunction, Covid-19, Depression, Long Covid, Post Covid-19 syndrome, Post SARS-CoV2, Postural orthostatic tachycardia syndrome, Pulmonary fibrosis

## Abstract

Long Covid is one of the most prevalent and puzzling conditions that arose with the Covid pandemic. Covid-19 infection generally resolves within several weeks but some experience new or lingering symptoms. Though there is no formal definition for such lingering symptoms the CDC boadly describes long Covid as persons having a wide range of new, recurring or sustained health issues four or more weeks after first being infected with SARS-CoV2. The WHO defines *long Covid* as the manifestation of symptoms from a “probable or confirmed” Covid-19 infection that start approximately 3 months after the onset of the acute infection and last for more than 2 months. Numerous studies have looked at the implications of long Covid on various organs. Many specific mechanisms have been proposed for such changes. In this article, we provide an overview of some of the main mechanisms by which long Covid induces end-organ damage proposed in recent research studies. We also review various treatment options, current clinical trials, and other potential therapeutic avenues to control long Covid followed by the information about the effect of vaccination on long Covid. Lastly, we discuss some of the questions and knowledge gaps in the present understanding of long Covid. We believe more studies of the effects long Covid has on quality of life, future health and life expectancy are required to better understand and eventually prevent or treat the disease. We acknowledge the effects of long Covid are not limited to those in this article but as it may affect the health of future offspring and therefore, we deem it important to identify more prognostic and therapeutic targets to control this condition.

## 1. Introduction

Following the emergence of the Covid-19 pandemic, the disease complications persisted in many post-Covid survivors long after acute infection (1, 2). This phenomenon is known as *long Covid* or *post-Covid-19* and soon spawned a series of studies on the long-term symptoms following acute Covid. The term “long Covid” was first generated in early 2020 by patients who suffered from symptoms of Covid-19 for weeks to months (3). This condition later led to the initiation of many support groups and campaigns for greater understanding, recognition, and treatment of "long-term Covid”.

**Figure 1:**
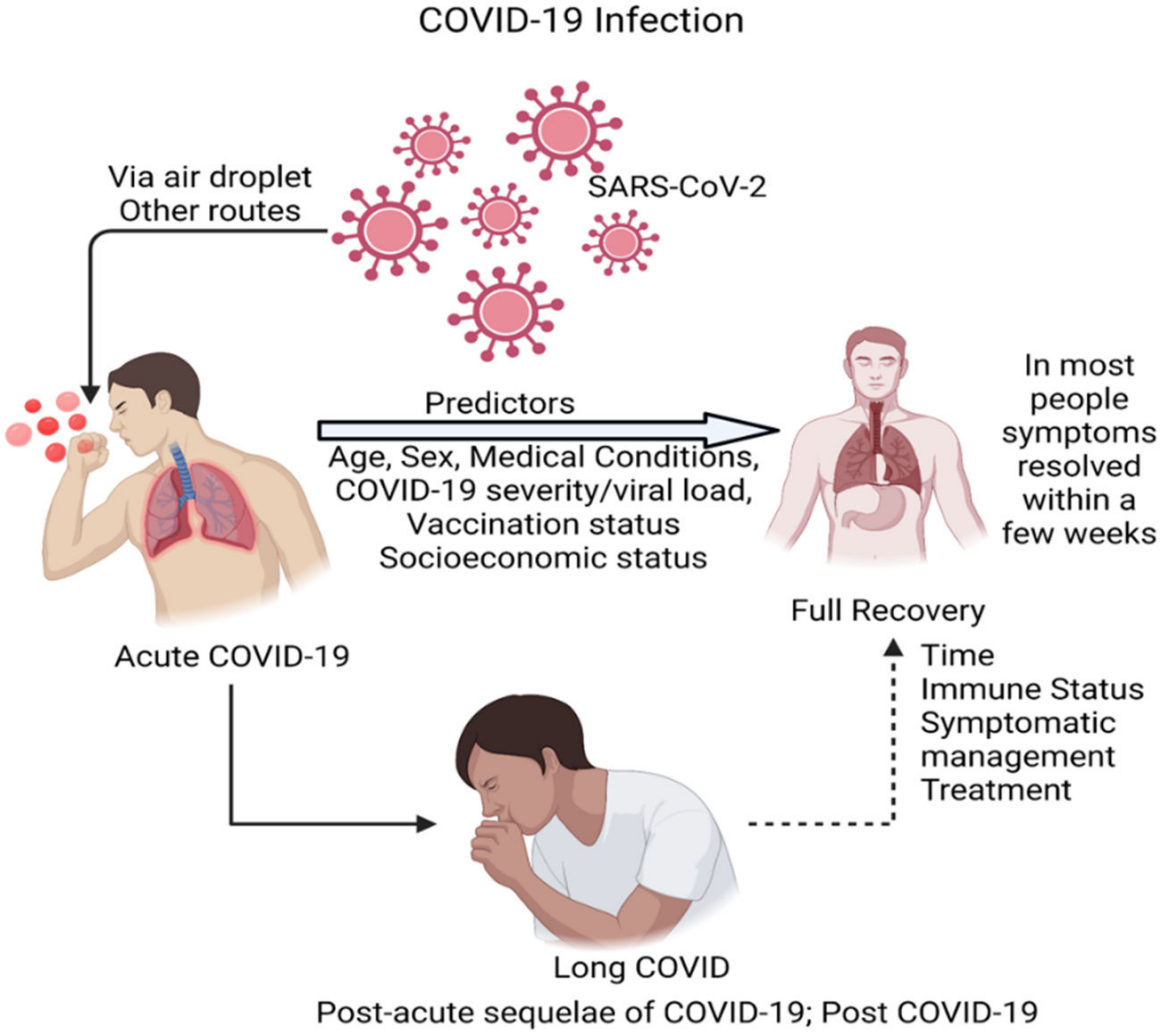
Long Covid as sequelae of acute Covid-19. Initial hypotheses regarding COVID-19 postulated that age, sex, preexisting comorbidities, and severity of Covid-19 can raise the likelihood of long Covid. However, subsequent studies suggest long Covid can occur regardless of prior comorbidities or severity of acute Covid-19.

It is important to note the difficulty of discerning whether post-Covid symptoms are a direct effect of Covid-19 or are due to other contributing factors (such as pulmonary fibrosis, medications used during acute treatment, intensive care syndrome, or exacerbation of a currently existing medical condition) (4) (Figure 1). Finding a clear boundary between such processes is often impossible in real patient scenarios. However, in this article, we regard long Covid as a diagnosis of exclusion to be made only when the elicited symptoms of the disease cannot be explained by another diagnosis such as post-intensive care syndrome or exacerbation of pre-existing health conditions.

It has been shown that Covid-19 infection can result in long Covid symptoms, regardless of prior health status or severity of initial acute infection (5). Many studies have shown that Covid -19 can persist in certain body tissues long past acute infection. It is known that the angiotensin converting enzyme 2 (ACE2) gene is expressed in various human tissues, and Covid-19 virus infection may occur via several routes, including air droplets, ocular transmission (6, 7). Therefore, Covid-19 can potentially have short-term and long-term effects on any of these organ tissues (8, 9). Studies have shown that chronic viral infections are associated with long-term fatigue syndrome (10). Although post-infectious disease syndrome (predominantly manifested by prolonged fatigue) has been a well-recognized phenomenon for the past 85 years, the considerably wider spectrum of long Covid symptoms begs for more research to understand the underlying pathophysiology of all long Covid symptoms. Current clinical trials are looking at the evolution of long Covid symptomatology and searching for more biomarkers to better understand the disease (NCT05610436, NCT05672602, NCT04964115, NCT05196516, NCT05172024, NCT05359159). Furthermore, the effect of treatment with Paxlovid during acute Covid-19 in the generation of long Covid symptoms is also being investigated (NCT05576662). In this review, discuss some of the organs affected by the disease, potential mechanisms contributing to pathophysiology, disease prognostics and current research on treatment options. We also review the effect of vaccines in the symptomology of long Covid.

## II. Long Covid symptoms, effect on organs, and pathological mechanisms

### A. General sequelae

**Figure 2:**
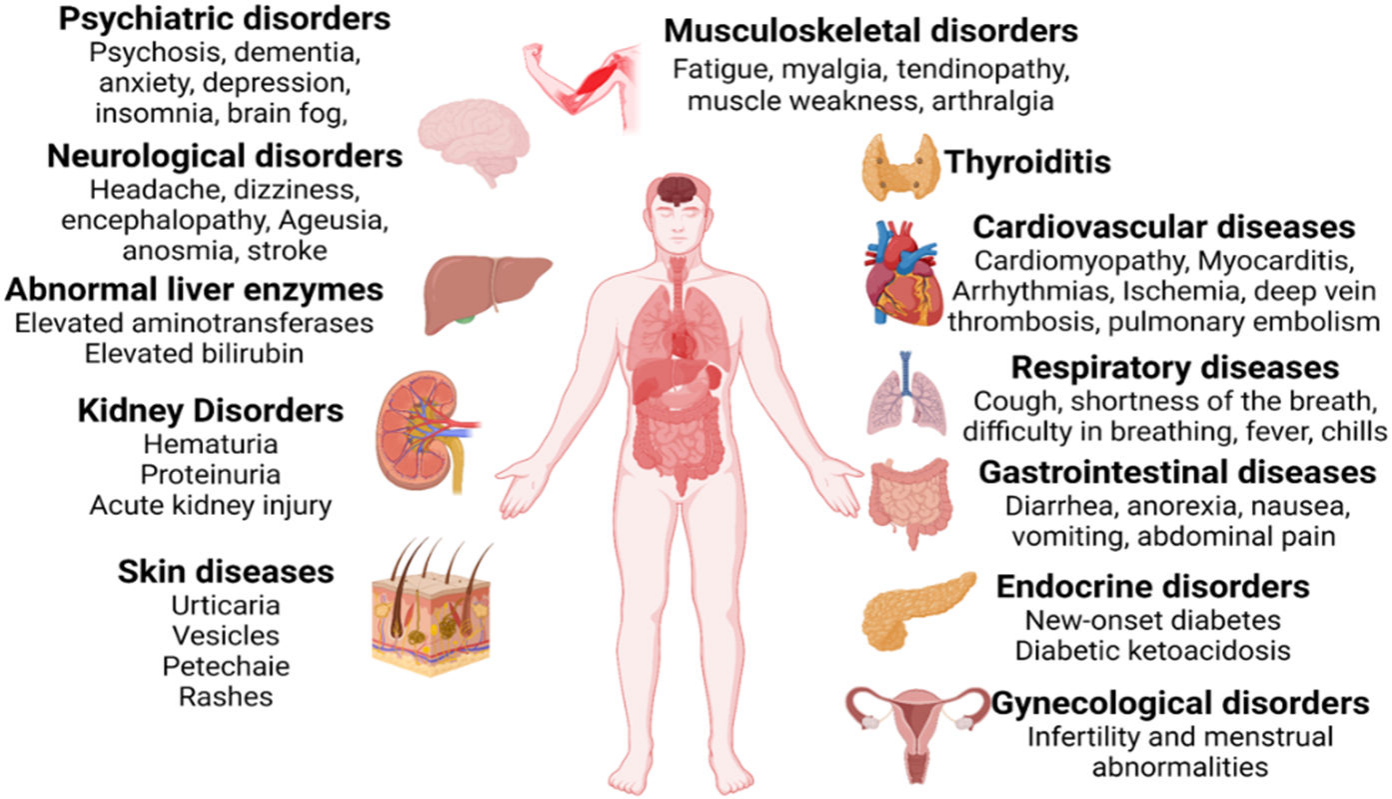
Covid-19 infection affects almost all organs and organ systems are affected resulting in different pathophysiology. Few of the key symptoms and outcome results are shown. This is primarily due to the sequelae of cytokine storm.

**Figure 3:**
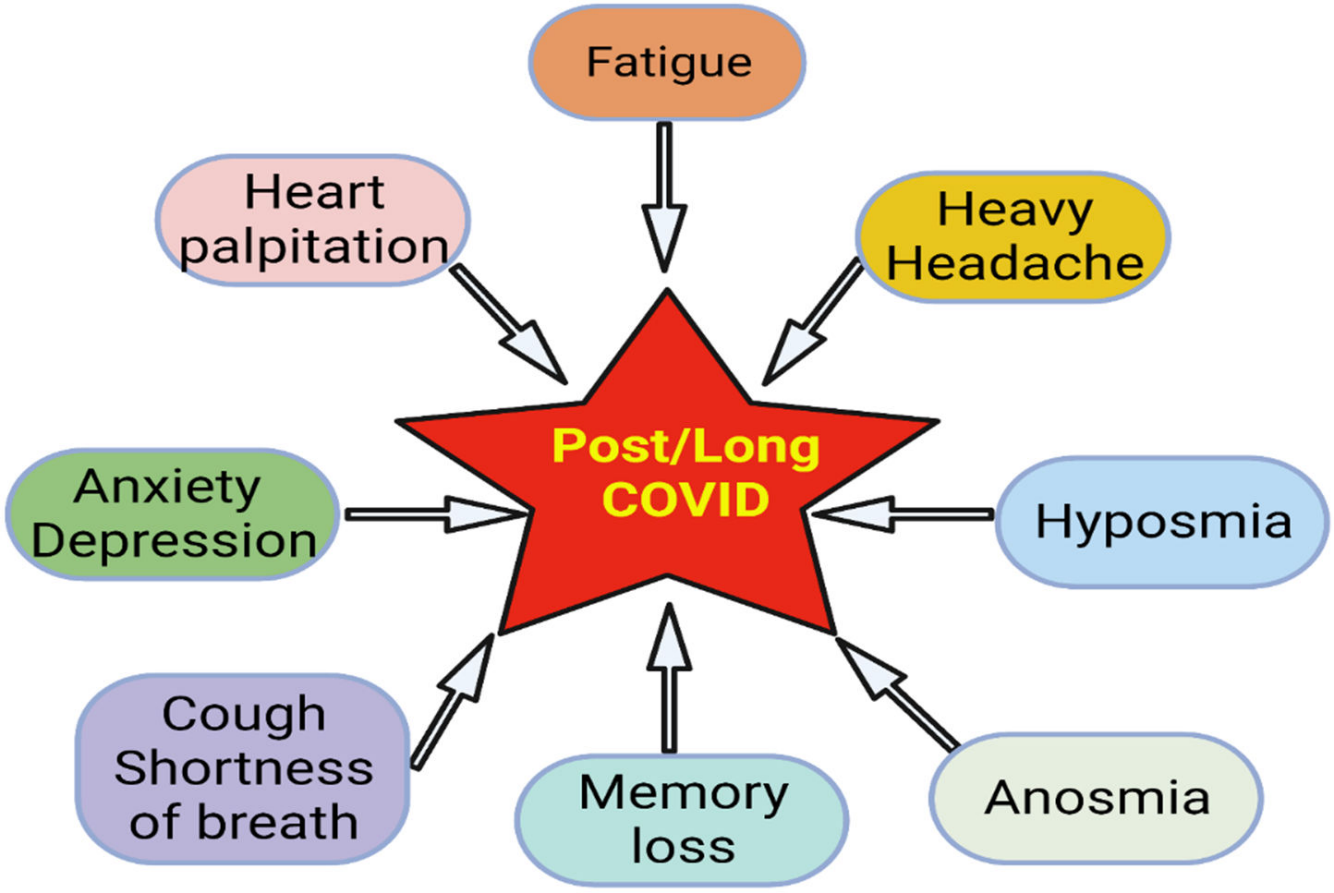
Major clinical complications in long Covid after 12 weeks of initial infection

Almost all organs and organ systems can be involved in Covid -19 infection, and many such complications can persist while others can reoccur (Figure 2). Some general symptoms of long Covid include fatigue (not improved with rest), heart palpitations, shortness of breadth, cough, anosmia, hyposmia, headache, cognitive dysfunction, parkinsonism, dementia delirium, “brain fog” (poor short-term memory, concentration, problem-solving, and executive function), mental fatigue, dizziness, vertigo, and anxiety/depression (Figure 3). Fatigue is the most reported symptom of long Covid irrespective of the severity of the infection (11, 12).

#### Effects in the Brain and Mechanisms of cognitive dysfunction and fatigue

**Figure 4:**
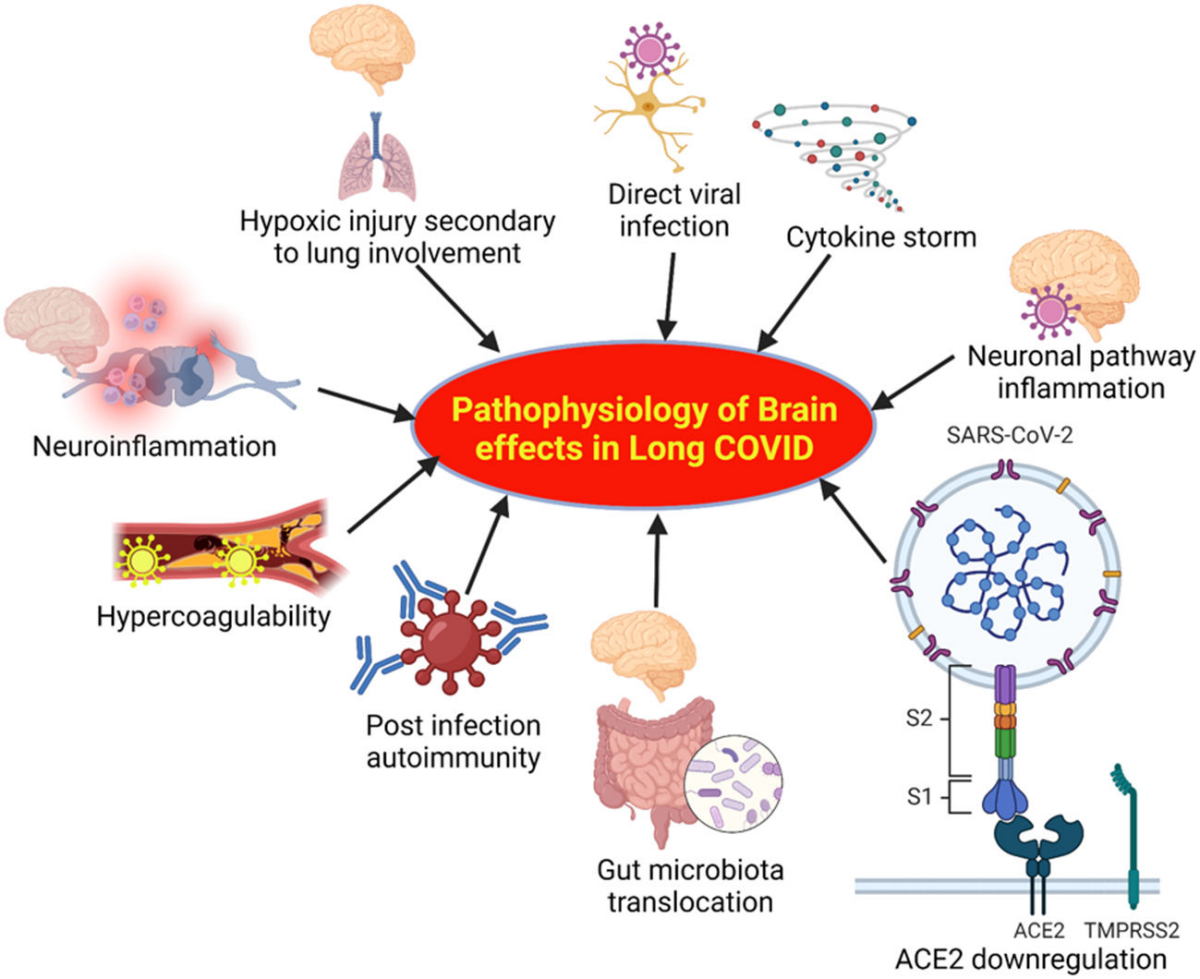
Major factors post Covid in the underlying pathophysiology of brain effects in long Covid.

The major factors underlying the pathophysiology of brain effects in long Covid are highlighted in Figure 4. These include direct viral infection, hypoxic injury secondary to lung involvement, cytokine storm, microglia and neuroinflammation, hypercoagulability, inflammation in the neuronal pathway, post infection autoimmunity, translocation of gut microbiota, and downreglation of ACE2 (7-9) (Figure 4). Effects of these factors are discussed below.

##### I Direct Invasion of the Central Nervous System (CNS):

ACE2 has been found in the neurons and glia of several regions of the CNS including the basal ganglia, hypothalamus, midbrain, pons, medulla, and cerebellum. This is thought to occur as SARS-CoV-2 can infect the CNS by binding to ACE2. Furthermore, the existence of ACE2 in endothelial cells theoretically is a portal for Covid-19 to enter and disrupt the blood-brain-barrier (BBB), thereby inducing injuries to the CNS (13). A recent clinical trial (NCT04851561) showed that long Covid fatigue is not only related to impaired cognition and decreased mood but also autonomic dysfunction at the level of limbic-vagal region in the CNS (14). One mechanism of this fatigue in long Covid is the invasion of the nucleus of the solitary tract (NTS). This nucleus includes most of the cell bodies related to the afferent cranial nerve 10 (CNX or vagus nerve) (13). Invasion of this nucleus contributes to long-term fatigue. This mechanism relates to the increase in specific inflammatory and infection-triggered cytokines (IL-6 and IL-10) that have been shown to lead to chronic fatigue syndrome (15). Nonetheless, in this process, the vagus nerve detects proinflammatory cytokines. Upon detection of these cytokines, the “sick behavior response” (including fatigue and sleep disturbance) is triggered in the dorsal brainstem which result in fatigue, myalgia, fever and other symptoms in both acute as well as long Covid (16).

##### II Microglia and neuroinflammation:

**Figure 5:**
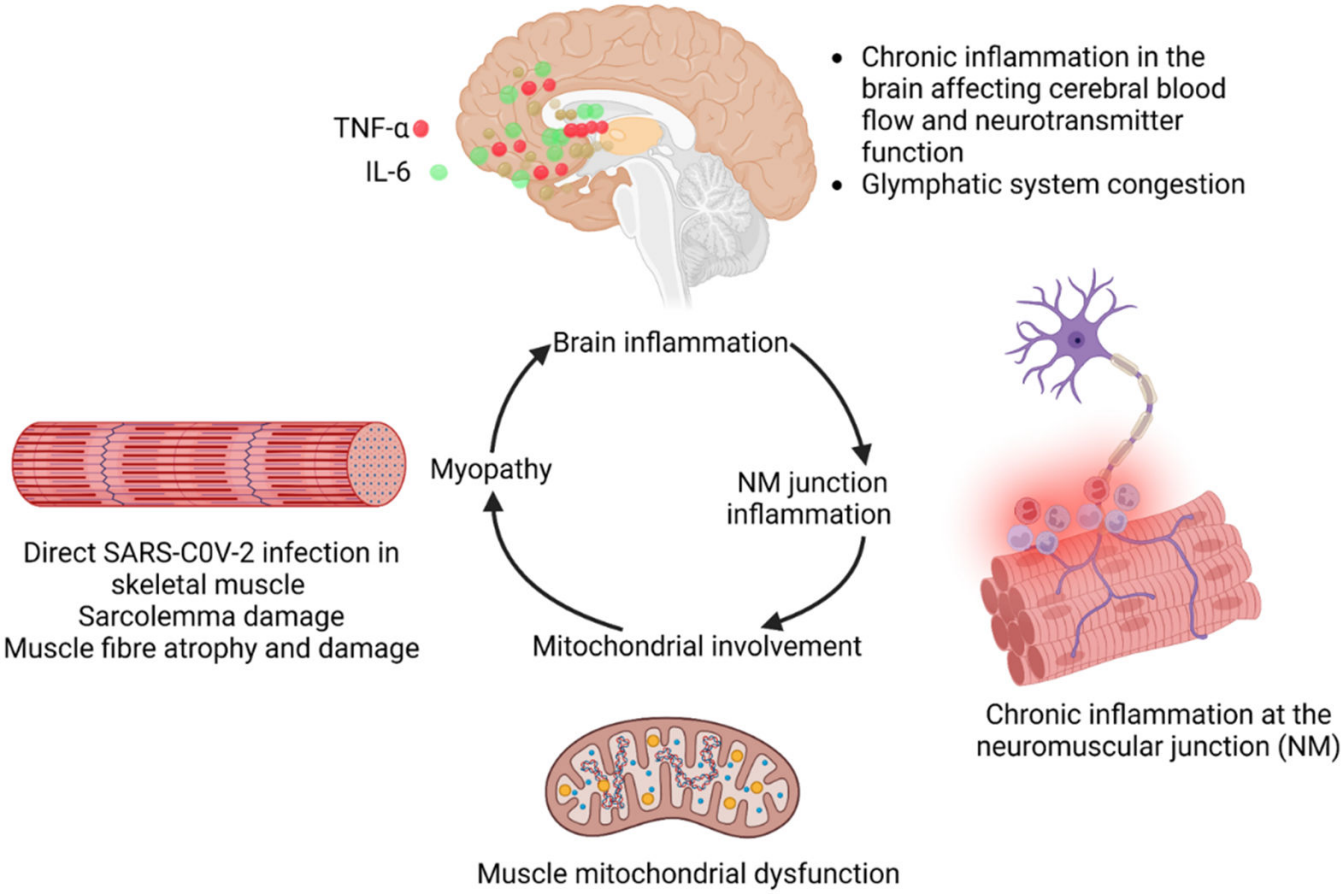
Potential mechanisms of the effect of SARS-CoV2 infection in the brain that induces chronic inflammation in the neuromuscular junction. This involves mitochondria leading to sarcolemma damage, muscle fibre atrophy and damage. All these result in myopathy.

It is known that systemic inflammation, defined as an increase in the level of key inflammatory cytokines IL-1β, IL-6, and TNF-α can induce neuroinflammation (Figure 5). It is also known that possible sequelae of systemic inflammation, immune and autoimmune responses, hypoxia, and thrombosis can result in neurological damage. Covid-19 has been associated with severe immune response, cytokine production, and systemic inflammation. Previous studies on systemic inflammation and sepsis have shown long-term decline in cognition and the initiation of myriad neurodegenerative diseases (13). It has also been postulated that chronic low-level brain inflammation caused by aging is another mechanism that can allow the persistence of symptoms of long Covid. It is, likewise, known that microglia play an important role in neuroinflammation (13). Thus it is hypothesized that Microglia also have important role in Covid-19-mediated neuroinflammation (13, 17). Furthermore, it has been demonstrated that these cells can also respond to stress through Cortisol Releasing Hormone (CRH) receptors they express. Furthermore, Covid-19 is known to affect the hypothalamo–pituitary–adrenal (HPA) axis of the corticosteroid pathway and induce stress. This pathway also affects immune modulation. Therefore, microglia can be activated in Covid-19 stress responses and feed into the neuroinflammatory responses during acute and long Covid (18).

##### III Autoimmune invasion of the CNS:

It has been shown that autoimmune responses generated due to long Covid can result in chronic fatigue (Figure 5). This is further explained in the autoimmune and autoreactive section, discussed below.

##### IV Congestion of cerebrospinal fluid (CSF):

One other pathophysiological mechanism for chronic fatigue syndrome post-Covid19 was introduced by Wostyn. based on previous studies on glymphatic system clearance and the changes in CSF outflow in Covid-19 patients (16). It is known that CSF drains into the lymphatic vessels through the cribriform plate. Covid-19 infection can cause the death of olfactory sensory neurons as well as blockage of lymph vessels in the cribriform plate when invading the olfactory bulb (19). Previous studies in mice have shown that such damage to the olfactory sensory neurons disrupts the CSF drainage through the cribriform plate (20). Further studies showed dysregulation of CSF drainage and blockage in CSF absorption overtime result in an increase in intracranial pressure (ICP). As consistent with this process, it is noteworthy in how many patients suffering from chronic fatigue syndrome also suffer from intracranial hypertension.

Our knowledge concerning increased Idiopathic Intracranial Hypertension in Covid-19 patients is limited to case reports and case series (21, 22). But congestion of CSF in the CNS not only leads to an increase in intracranial pressure but also results in accumulation of toxins within the CNS. These mechanisms can, therefore, lead to chronic fatigue syndrome in Covid-19. Current clinical trials are underway to clarify mechanisms of chronic fatigue syndrome and long Covid better understand the more detailed disease dynamics (NCT04806620, NCT04883190, NCT05323318). One clinical trial assesses the overall effects of long Covid on the CNS at one year follow-up (NCT05492292). Another is a study of damage in the brain of patients suffering from long Covid (NCT05433324).

###### Treatment:

Muller, Thomas, et al. suggested that treatment of fatigue, chronic exhaustion, and cognitive impairment induced by long Covid can be achieved through the usage of amantadine, an antiviral medication also used in Parkinson’s disease, and memantine, an acetylcholine esterase inhibitor used in Alzheimer’s disease. Further clinical trials are required to confirm such hypotheses (23). Currently, a clinical trial is studying the effect of vortioxetine, an SSRI on long Covid cognitive deficit (NCT05047952).

If it is proven that one of the mechanisms underlying chronic fatigue is related to CSF congestion and issues with lymphatic drainage in long Covid, lymphatic drainage and cranial techniques may be a possible early non-invasive therapies for post-Covid19 fatigue. Lymphatic drainage is a technique research studies have demonstrated to improve lymphatic congestion and waste removal from the brain. A few studies have shown this technique improves symptoms of fatigue in long Covid patients (24). Expanding cohorts within these and similar studies will further help shed light on mechanisms and treatment of chronic fatigue syndrome of long Covid.

Moreover, it has been demonstrated that chronic fatigue of long Covid can be alleviated using high-dose intravenous vitamin C for its anti-inflammatory, antioxidant, and immunomodulatory properties (25). Furthermore, studies conducted on non-invasive brain stimulation have shown promising results in reducing fatigue and anxiety in long Covid (26). Currently, clinical trials are measuring and investigating more protocols to enhance the use of this treatment in long Covid patients (NCT05289115, NCT05289128).

### B. Psychiatric sequelae

Some of the psychiatric symptoms associated with long Covid include anxiety, depression, post-traumatic stress disorder (PTSD), brain fog, psychosis, sleep disturbance (unrefreshing sleep, exhaustion, vivid dreams, or nightmares). PTSD, depression, and brain fog have been recognized as some of the prevalent symptoms of long Covid and Severe Acute Respiratory Syndrome (SARS)-CoV family (27). Studies have shown symptoms of fatigue, anxiety, depression, and sleep difficulties to be the sequelae in some of the Covid-19 infection survivors (28). A clinical trial is looking at the neuropsychiatric sequelae of Covid-19 (NCT05615415). However, it is quite likely that additional clinical trials are warranted to further examine the neuro[sychiatric disorders in long Covid.

#### Mechanisms of fibromyalgia

##### I. High inflammatory cytokines:

As will be discussed in detail in the Mast Cell Activation Syndrome (MCAS) section, brain fog found in long Covid mimics the chemofog and MCAS symptoms due to elevation of neuroinflammation and immune signals caused by IL-1, IL-6, and TNF-α in Covid-19 (29). These cytokines can also altere CRH, Adrenocorticotropic Hormone (ACTH), and cortisol levels in the HPA axis and lead to chronic symptoms of fatigue and fibromyalgia-like disease picture (30). IL-6 has also been shown to cause muscle loss through disrupting metabolic homeostasis in musculature (31).

##### II. Direct invasion of skeletal myocytes:

ACE2 has been found to be expressed in skeletal muscles in a manner similar to cardiac muscles (32). Therefore, it can be hypothesized that Covid-19 can directly attack skeletal myocytes and lead to long Covid fibromyalgia symptoms (33). The potential underlying effects are shown in Figure 5 whereby chronic inflammation in the brain affects cerebral blood flow and the function of neurotransmitters and induction of the congestion in glymphatic system. Chronic inflammation in the neuromuscular junction involves mitochondria leading to sarcolemma damage, muscle fibre atrophy and damage, resulting in the development of myopathy (Figure 5). Currently, a clinical trial is looking at the musculoskeletal pain associated with long Covid (NCT05358119).

#### Mechanisms of depression

##### I. Highly inflammatory cytokines:

Several systemic reviews and meta-analysis studies on the correlation between cytokines level and depression, anxiety, and PTSD showed evidence of higher levels of IL-1, IL-6, and TNF-α in individuals with Major Depressive Disorder (MDD), anxiety, and PTSD compared to controls (34). Studies have also shown that cytokines affect the dopaminergic pathways in the CNS leading to anhedonia, fatigue, and impaired cognition in primates (35). Several studies have also shown that elevated cytokines contribute to depression and anhedonia in humans (36). Similarly, these processes can affect mood regulation by altering serotoninergic and norepinephrinergic pathways.

One hypothesis about inflammatory responses causing a long-term effect in the CNS derives from cytokines can storm during severe Covid-19. It is postulated that cytokine storm activates indoleamine 2,3-dioxygenase (IDO-1) which increases kynurenine metabolism (8). Kynurenine has extensive physiological roles, including immune regulation, inflammation, and neuroprotection. Kynurenine is also involved in the production of chemokines that can lead to long-term brain impairment with prolonged exposure affecting the HPA axis. This increases glucocorticoid release, which initially suppresses the immune system and has a protective role.

However, prolonged exposure to chronic stressors of severe or prolonged infection as well as prolonged cortisol release impairs and desensitizes glucocorticoid receptor function and reduces the immune system response to cortisol release. This in turn leads to the accumulation of high levels of cytokines and contributes to depressive symptoms (37). Furthermore, these pro-inflammatory cytokines also induce sickness behaviors (including sleep disturbance, loss of appetite, and fatigue) that have symptoms overlapping with depression (38). At the same time, Covid-19 increases the level of angiotensin II through the ACE2 pathway, leading to a further increase in kynurenine and the production of pro-oxidative and pro-inflammatory metabolites, that further exacerbate cognitive dysfunction and lead to signs of long-term depression.

##### II) Direct attack on the CNS:

Covid-19 has been shown to invade the CNS of affected individuals through ACE2 receptors without significant signs of damage from any Covid-19-induced hypoxia (6, 39, 40). Some post-covid studies have also shown signs of CNS pathologies in patients with negative Covid-19 PCR (41). Covid-19 can also have long-term damage to the brainstem through immune activation of leukocytes, microglia, and astrocytes while causing microthrombosis in the brainstem vasculature as well (31, 41). Furthermore, direct Covid-19 attack to the CNS or its vascular endothelia can directly damage these tissues as well as dopaminergic, serotonergic, and norepinephrinergic neural pathways embedded in the brainstem. Injuries to these regions lead to impaired emotions and cognition which may in pat explain the depressive, anxiety, and fatigue symptoms of long Covid. Moreover, dopaminergic neurons damage in can result in anhedonia, reduced cognitive capacity and fatigue in long Covid. Overall, knowing that neuroregeneration is a slow process in humans, injury to the CNS during Covid-19 can potentially result in a large spectrum of neurocognitive symptoms during long Covid (Figure 4).

#### Mechanisms of sleep disturbance:

##### I. Highly inflammatory cytokines and impaired blood brain barrier:

It is hypothesized that the accumulation of cytokines and their passage through the blood brain barrier (BBB) in circumventricular areas of the CNS, such as the hypothalamus, leads to autonomic dysfunction that can result in poor sleep/wake cycles and cognitive impairment (42). Damage to endothelial cells increases leakage and further exacerbates BBB disruption and peripheral immune cell invasion.

##### II. Direct attack to the brainstem:

As was discussed earlier, Covid-19 can directly invade the brainstem through ACE2 receptors. The brainstem is known to control the sleep–wake cycle (43). Therefore, part of the long-term sleep disturbance post-Covid-19 may be due to damage to this important structure.

###### Treatment:

Data suggest that early use of antidepressants during Covid-19 infection can reduce the risk of clinical deterioration, although no official recommendation for or against antidepressants use has yet entered current Covid-19 protocols (44, 45, 46, 47). Aside from any direct effects to do with long Covid mechanisms, antidepressants have the beneficial effect of reducing depression (48). Interestingly, several hypotheses posit multifactorial features of Selective Serotonin Reuptake Inhibitor (SSRI) antidepressants may be beneficial in Covid-19 and long Covid. One hypothesis notes the high expression of the serotonin transporters in human lungs (49). The vasoconstrictive role of serotonin in lung endothelium is regulated by serotonin transporters (50). Therefore, utilizing SSRIs in Covid-19 lung infection may potentially benefit lung function. Another hypothesis focuses on the affinity of some SSRIs (such as fluvoxamine, citalopram, sertraline, and fluoxetine) for an endoplasmic reticulum protein, the Sigma-1 receptor (S1R). S1R activation has been shown to interfere with the initial steps in viral RNA replication (51). It is postulated that SSRIs can potentially have a prophylactic role in reducing viral RNA replication through the activity of S1R (44).

Other scientists investigated the role of SSRIs in inhibition of acid sphingomyelinase (ASM). ASM has a significant role in bacterial and viral infection, facilitates ceramide production and viral entry into the cell (52). It has been shown that antidepressants including some of the SSRIs (fluoxetine, sertraline, paroxetine) and the tricyclic antidepressant (amitriptyline) can be included as part of a larger group comprised of functional inhibitors of acid sphingomyelinase (FIASMAs) that inhibit ASM activity and reduce Covid-19 infection (53).

Interestingly, another SSRI, fluvoxamine, increases the level of melatonin by inhibiting CYP1A2, a Cytochrome P450, enzyme. It is known that melatonin, produced naturally by pineal gland, not only regulates sleep but also reduces inflammation and oxidative stress. Therefore, it is hypothesized that fluvoxamine reduces Covid-19 and its sequelae by increasing this hormone in the body (54). Future studies might usefully investigate the possible role of melatonin in the treatment of depressive symptoms during long Covid (55).

It is postulated that SSRIs, through activation of S1R as well as neutralizing IDO-mediated inflammation, result in activation of anti-inflammatory pathways and therefore downregulate cytokine production (56). This reduces systemic as well as neuroinflammation which may reduce signs and symptoms of MDD during both acute Covid-19 and long Covid (57). Nevertheless, it is still important to be cautious about using antidepressants in pregnant women with Covid-19, since increasing evidence associates SSRIs use during pregnancy with the risk of autism spectrum disorder in offspring (58). Studies of natural flavonoids as promising, and possibly safer, alternatives possible anti-neuroinflammatory medications.

Natural flavonoids such as luteolin and quercetin can inhibit viral entry into host cells and inhibit neuroinflammation. Preliminary studies on the effect of luteolin in patients with long Covid syndromes of cognitive difficulty and fatigue after mild Covid-19 showed restoration of GABA activity and cortical plasticity after 8 weeks of luteolin treatment (59). It has also been shown that TNF-α antagonists may be new therapeutic agents for MDD (60). Therefore, this option can be explored in MDD caused by systemic and prolonged inflammatory responses during long Covid.

Another small study found a reduction in Covid-19 inflammation using lithium salt, a mood stabilizer and anti-depressant (61). Future studies and clinical trials can further explore whether lithium reduces inflammatory and depressive symptoms of acute and long Covid. A single case report showed reduced depressive symptoms of long Covid in a patient using transcranial direct current stimulation (62). Moew auch studies are yo be further welcomed in the future. A clinical trial is also exploring the use of temelimab as a Disease Modifying Therapy (DMT) for neuropsychiatric symptoms of long Covid (NCT05497089). Lastly, a clinical trial is currently investigating the role of glutamatergic modulation in the treatment of long Covid depressive symptoms (NCT05690503).

### C. Ear, Eyes, Nose and Throat

Other symptoms of Covid-19 include tinnitus, vertigo, anosmia, phantosmia (persistent disagreeable background smell) or parosmia (distorted sense of smell) (11, 63, 64). It has been shown thatolfactory dysfunction persisted in some Covid-19 patients for close to 6 months post-Covid-19 recovery (65). One study of post anosmia was able to show an excellent prognosis of anosmia recovery at 1-year post-Covid-19 infection (66). While tinnitus and vertigo have been reported to be highly prevalent in long Covid, self-reported hearing loss has been less frequent. Some studies also shown no evidence of long-term pathophysiological processes causing loss of hearing in long Covid (64, 67). Nonetheless, when considering sensory deficits during long Covid, it is also important to note that some Covid-19 treatments, especially quinines such as hydroxychloroquine used early in the Covid-19 pandemic, or other cinchona alkaloids, have “cinchonism” side effects (68). Cinchonism is known to result in headache, tinnitus, nausea, vomiting, dizziness, flushing, and hearing loss as well as cardiac arrest and convulsion in severe cases. Furthermore, chloroquine/hydroxychloroquine may lead to symptoms of xerostomia in the oral mucosa which can be treated with zinc supplementation. Therefore, obtaining a thorough medical history is a key to understanding the possible causes of the sensory symptoms in the post-Covid-19 survivors. Furthermore, azithromycin, used as anti-inflammatory in some treatment protocols, as well as some of the antiviral agents and protease inhibitors during Covid-19 have an ototoxic side effect (69).

#### Mechanisms of vestibulocochlear disorders

##### I. Direct attack on the vestibulocochlear system

It is hypothesized that vestibulocochlear system damage is due to direct viral entry through blood vessels, lymphatics, meninges, and nerves (70). Tinnitus and sensorineural hearing loss observed due to Covid-19 patients is thought to be induced by direct viral attack and damage of hair cells of the inner ear as further evident by reduced amplitude of transient otoacoustic emissions as well as finding virus in the middle ear and mastoid of patients with Covid-19 (71).

##### II. Inflammatory-induced damage

Covid-19 is known to cause long-term and persistent inflammatory responses in patients with long Covid. Studies have shown inflammatory responses and increased cytokines harm the vestibulocochlear apparatus. Signs of inflammation in inner ear vessels and the stria vascularis were found (72, 73). It is hypothesized that this mechanism maybe cause tinnitus in Covid-19 and long Covid-19 (74).

###### Treatment:

Few studies have been completed in the treatment of long covid vestibulocochlear symptoms, a pilot randomized controlled trial looked at the safety and feasibility of using transcutaneous auricular vagus nerve stimulation for management of these symptoms. Also mentioned previously, several clinical trials are investigating optimal protocols required for brain stimulation treatments (NCT05289115, NCT05289128).

#### Mechanism of olfactory disorders

##### I. Direct attack on the CNS and olfactory bulb:

It has been shown in mice transgenic for human ACE2 that SARS-CoV can enter the CNS through inoculation of the olfactory bulb (6, 19, 75). Interestingly, when mice were inoculated with a low dose of SARS-CoV, virus was only detected in the brain and not the lungs (75). Furthermore, small studies have shown that patients with Covid-19 infection self-reported symptoms of anosmia and phantosmia, implying that the olfactory bulb is involved in the disease (76, 77). Further studies in humans are required to assess if the disease is spreadto the CNS through this transnasal mechanism.

##### II. Autoimmune attack on the CNS and olfactory bulb:

Antibodies that are produced against Covid-19 can cross-react with proteins in multiple tissues of the body including the olfactory bulb. Such reactions can potentially cause damage to the bulb and lead to symptoms of anosmia, phantosmia or parosmia in the long-term (78, 79).

###### Treatment:

Use of luteolin (a flavonoid) can reduce the symptoms of olfactory dysfunction and impaired memory in patients suffering from long Covid (80). This effect is mediated through mast cell inhibition, as well as inflammatory cytokines (TNFα and IL-1), chemokine CCL2 and CCL5 secretion inhibition (81). Moreover, two clinical studies are currently looking at the effect of fluvoxamine, a selective serotonin reuptake inhibitor used for treatment of obsessive-compulsive disorder, for recovery of parosmia in long Covid patients (NCT05216614).

Other studies suggest use of anti-inflammatory agents such as systemic steroids, oral and topical corticosteroids for treatment of long Covid olfactory impairment. Studies to determine the effectiveness of corticosteroid sprays are ongoing. Nonetheless, little to no side effects were foreseen in short term therapy which suggests the potential benefit may outweigh risk of usage. Furthermore, use of nasal calcium buffers, nasal sodium citrate, phosphodiesterase inhibitors and nasal vitamin A for olfactory nerve regeneration may also be effective (82).

Olfactory training has been reported as a non-pharmacological treatment for long covid. It is hypothesized that olfactory training is caused by regeneration of olfactory receptor neurons and improvement in olfactory information processing (83). This treatment includes exposure to high intensity odors in a short period of time over several months.

#### Mechanisms of optic disorders

##### I. Direct attack:

ACE2 receptor is expressed in the retina, choroid, and ciliary body (6). In one postmortem study of Covid-19 patients, the virus was isolated from intraocular tissues and ocular surfaces (84). It is hypothesized that direct viral attack can result in some visual disturbances in long Covid.

##### II. Choroidal vascular and thickness shrinkage:

Human choroidal vessels provide nutrients and oxygen to the retina and photoreceptors. A recent study looked at changes in choroidal thickness. According to this study, choroidal thickness significantly decreased in all layers . In longitudinal follow-up at post-infection month nine, these patients had persistent choroidopathy to reverse and recover (85).

##### III. Molecular mimicry and thromboembolic events:

Certain antigens in Covid-19 have molecular mimicry to retinal structure that can induce autoimmune responses and thrombosis in ocular blood vessels (6, 72, 86). Current studies are directed to the retinal microvasculature in patients with long Covid syndrome (NCT05635552).

###### Treatment:

Limited studies are available regarding treatment for long Covid visual deficit. However, one report of non-invasive brain microcurrent stimulation in two long Covid patients with visual deficits found reduced vascular dysregulation and improved visual acuity (87). Further studies are required to confirm these observations.

#### Mechanisms of gustatory disorders

##### I. Direct attack:

The mucous membranes of the oral cavity expresse ACE2 receptors (88). Furthermore, Covid-19 binds to sialic acid receptors on gustatory cells resulting in their increased sensitivity threshold causing loss of the sense of taste (89). Damage to salivary glands due to Covid-19 infection requires several months to recover. Also, Covid-19 neuroinvasion may lead to dysfunction of gustatory and olfactory neurons. Given that the function of sense of smell and taste are linked together, the loss of one will impair the function of the other.

##### II. Zinc deficiency:

During long Covid, salivary damage may persist for a long period of time. Therefore, it is likely patients experience xerostomia, especially with prior exposure to chloroquine/hydroxychloroquine or low zinc levels. Zinc receptors in submandibular gland regulate salivary secretion. Furthermore, many patients with Covid-19 infection have low zinc levels (90). Zinc has a major role in immune modulation and therefore, individuals with zinc deficiency are more prone to infections. Thus individuals with long Covid who have persistent gustatory deficits and low zinc levels are highly likely to develop xerostomia.

###### Treatment:

As the olfactory and gustatory function are linked together, it can be hypothesized that improving olfactory deficits in long Covid can also improve dysgeusia. Neurostimulation with ATP, B vitamins and steroids have been postulated to improve signs of gustatory dysfunction in Covid-19 patients (91). Patients with persistent long Covid gustatory deficits often have low zinc levels. Hence, zinc supplementation be a potential treatment for gustatory xerostomia in long covid gustatory disorder (90). Nonetheless, further studies are required to determine more mechanisms and treatment options for gustatory deficits of long Covid.

### D. Pulmonary sequelae

Exertional breathlessness, reduced exercise tolerance, altered breathing, chest pressure, chest pain, dyspnea, cough are some of the known cardiopulmonary sequelae of long Covid. Overall, there are two categories of long Covid cardiopulmonary sequelae. The first is persistent complications from severe acute phase injuries due to severe inflammatory responses and treatment in the intensive care setting (92). This first category is a complex picture given that the symptoms and course can be caused by tissue injury in the acute setting, mechanisms of long Covid, or both. The second category, on the other hand, is persistent symptoms of Covid-19 without signs of organ damage. This is a simpler scenario with processes mainly driven by long Covid. Interestingly in this category, initial Covid-19 disease severity does not determine severity of long Covid (28). Nevertheless, in both categories, exertional breathlessness and excessive breathing can be seen (93). Currently, a clinical trial is looking at the sequelae of interstitial lung disease in long Covid (NCT05514522).

#### Mechanism of lung disease

##### I. Highly inflammatory cytokines and tissue injury:

Persistent cardiorespiratory symptoms post-severe Covid-19 and intensive care discharge are contributors to long Covid symptoms due to the lung injury during acute Covid-19. Such injuries impair residual lung function (94). In the initial invasion of Covid-19 into the lung tissue, the virus binds to ACE2 receptors n lung endothelial cells causing injuries. Such injuries lead to recruitment of inflammatory cells and cytokine release. Chronic inflammation is related to prolonged elevation of severe Covid-19 inflammatory cytokines IL-1β, IL-6, and TNF-α as well as IL-8 and reactive oxygen species. These proinflammatory cytokines result in a long-term profibrotic state leading to a tissue environment that is prone to collagen deposition and fibrotic changes (95).

##### II. Microangiopathy and autonomic dysregulation:

In the acute phase infection, initiation of thrombosis and intravascular coagulation mechanisms lead to further tissue damage and microangiopathies (8, 96). These microangiopathies have been postulated to cause dysregulation of autonomic control of both chemo- and mechanoreceptors. Treatment for these autonomic dysregulations will be further discussed in the “autonomic” section of this review. The mechanisms proposed for reduced exercise tolerance, exertional hyperventilation, and breathlessness can partly originate from the autonomic dysregulations that impede ventilatory control. Currently, a clinical trial is looking at the prognostic of dysfunctional breathing in long Covid (NCT05217875).

##### III. Immunothrombosis and elevated D-dimer:

While the immunothrombosis nature of acute severe Covid-19 is known, studies are investigating mechanisms underlying exertional breathlessness of long Covid. Studies have shown that while many coagulation parameters downtrend during Covid-19, D-dimer remains elevated in patients with symptoms of breathlessness with no signs of pulmonary embolism upon CT angiogram (97). The role of elevated D-dimer in the cardiopulmonary effect of long Covid is also being investigated.

##### IV. Neuroinflammation:

It is also hypothesized that chronic cough of long Covid with no signs of fibrotic damage to the lung parenchyma or airway damage might be caused by Covid-19 invasion of vagal sensory neurons and/or neuroinflammatory responses (7, 13). Neuroinflammatory mediators can affect the vagus nerve at any or all of the level of the nerve terminal in the airways, lung tissue or in the axons, cell bodies, or at vagal sensory center in the CNS. These invasive mechanisms lead to hypersensitivity of the central and peripheral cough pathways. Further investigation is required to explore this hypothesis.

###### Treatment:

The initial recommendation to reduce exacerbation of dyspnea is to self-manage through avoidance of smoke, pollutants, extreme temperature changes, and intensive exercise. Other management strategies rely on continuous breathing exercises, pulmonary rehabilitation, optimal postural positioning, and the controlled use of opioids for the treatment of dyspnea (98).

In cases of pulmonary fibrosis in long Covid, selected antifibrotic therapeutics are recommended in severe cases (99). Lung transplant may be an option in individuals with advanced disease progression and minimal comorbidities. Another treatment being tested for lung inflammation in Covid-19 and long Covid is cromolyn. This is a mast cell stabilizer that inhibits release of chemokines (100). Likewise, the use of Janus kinase (JAK) inhibitors (including tofacitinib, ruxolitinib, baricitinib) to regulate the immune system in long Covid lung symptoms is also being investigated (101). Currently, there are also other clinical trials exploring the efficacy of using hyperbaric oxygen, and montelukast to treat respiratory symptoms of long Covid (NCT04695704) (102, 103). For chronic cough, the use of neuromodulators such as gabapentin or opiates are often used empirically in addition to anti-inflammatory medications (104).

### E. Heart and vascular

Some of the cardiovascular symptoms of long Covid are chest pain, myocarditis, pericarditis, microvascular angina, cardiac arrhythmia, including inappropriate sinus tachycardia, atrial flutter, atrial fibrillation, palpitation, and high burden of ventricular ectopy. Chest pain, palpitation, and impaired pulmonary diffusion capacity after six months are common cardiopulmonary symptoms of long Covid (8, 72, 105). Individuals with long Covid may have abnormalities on cardiac imaging tests, such as echocardiography or MRI, even if they do not have cardiac symptoms in the acute setting (106).

#### Mechanisms of cardiovascular disease

##### I. Direct invasion of cardiomyocytes:

Covid-19 can directly infect the heart through the ACE2 receptors (8). Covid-19 infection can cause damage to the sarcomere and lead to the enucleation of cardiomyocytes (107, 108). Such events can induce prolonged inflammation, cellular damage, cardiac myositis, and fibrosis. Furthermore, Covid-19 can lead to symptoms of cardiovascular abnormalities even in those at low risk of severe Covid-19 (109).

##### II. Metabolic alterations:

Wu and colleagues (110) reported on 25 SARS patients 12 years post-recovery from SARS infection. Compared to healthy controls, the SARS group showed significantly higher cardiovascular abnormalities but normal pulmonary ventilation function. Possible mechanisms of this difference may have to do with systemic metabolic alterations and increased heart metabolic demand. These may loosely trace back to steroid treatment,consequential reduction in cardiac reserve, and changes in the renin-angiotensin-aldosterone system (RAAS) rather than the viral invasion itself. Furthermore, changes in lipid profile due to altered metabolism increases risk of cardiovascular and stroke in these patients long term (110). We postulate that similar long-term mechanisms can occur in long Covid.

##### III. Increased cardiac demand:

In the early stages of Covid-19 infection, elevations of both troponin and Brain Natriuretic Peptides (BNP) occur, which indicates cardiac stress, increased demand, and injury to the cardiovascular system. Such injuries can lead to fibrosis and scarring of the heart, sometimes inducing cardiomyopathy and arrhythmia, manifested as palpitations in patients with long Covid-19 (111). Myocardial inflammation caused by Covid-19 can also lead to myriad cardiac complications ranging from myocarditis to cardiac arrhythmias, heart failure, acute coronary syndrome, or sudden cardiac death (8, 112).

##### IV. Acute medication toxicity:

Another possible contributor to cardiac sequelae of post-acute Covid-19 may be medication related. Especially early in the pandemic, when the clinical trials were ongoing and guidelines were uncertain, several antiviral therapies were provided to patients. M antivirals such as interferon confer risk of cardiac toxicity and can otherwise adversely affect the cardiovascular system in the long term . While initial studies of interferon and combination therapies showed improvement in acute Covid-19 symptoms, later studies found no difference in hospitalized Covid-19 patients (113, 114). Therefore, current guidelines recommend against the use of interferons alpha, beta, and lambda for treatment of hospitalized Covid-19 patients. However, follow-up studies of patients treated with interferons for acute Covid-19 infection for cardiotoxicity could shed light on the plausibility of this hypothesis.

##### V. Autoimmune attack:

As further discussed in the “SARS-COV-2 specific and autoreactive immune responses” section, antiphospholipid antibodies produced in some cases can lead to vascular inflammation (115). Such processes lead to damage in the large vessels which jeopardizes vasculature health in post-covid survivors (8, 116). Furthermore, autoantibodies produced against nociception receptor, β_2_-adrenoceptor, α_1_-adrenoceptor, endothelin receptor, muscarinic receptor, angiotensin II Type 1 receptor, and MAS receptor in post-Covid patients are hypothesized to contribute to neurologic, autonomic, and cardiovascular symptoms of long Covid (117).

###### Treatment:

β-blockers may be helpful in the treatment of cardiovascular symptoms of long Covid. Furthermore, myocarditis sometimes can resolve with supportive care and immunomodulating therapy (118). Further studies are required to better determine other routes for cardiovascular care in patients with long Covid symptoms.

### F. Autonomic system sequelae

Palpitation, dizziness, orthostasis, gastrointestinal disturbances, generalized pain are some of the autonomic symptoms seen during long Covid. Infection is one of the most prevalent triggers for new onset orthostatic intolerance and postural tachycardia syndrome (119). Studies report cases of post-covid autonomic syndrome with symptoms of orthostatic intolerance, dizziness, brain fog, and postural orthostatic tachycardia syndrome, as well as peripheral nervous system dysfunction (120).

#### Mechanisms of autonomic dysfunction

##### I. Autoimmune attack:

It is speculated that autoimmune mechanisms and autoantibody formation may be involved in the manifestation of autonomic symptoms in long Covid (121). It has also been shown that postural tachycardia syndrome and orthostatic hypotension are associated with these processes (122). As also will be discussed in the “SARS-COV-2 specific and autoreactive immune responses” section, autoantibodies produced against β_2_-adrenoceptors, α_1_-adrenoceptors, and muscarinic receptors also affect the autonomic nervous system. This can lead to autonomic dysregulation with downstream effects observed in long Covid (117). Currently, a clinical trial is looking at further mechanisms of post-Covid-19 tachycardia syndrome (NCT05421208).

##### II. Direct attack of the brainstem:

Some symptoms of dysfunctions due to brainstem damage are very similar to those of long Covid. For instance, one of the major roles of the brainstem is to regulate the cardiorespiratory center. As was noted in the general fatigue section, fatigue caused by long Covid is partly attributed to invasion of the NTS in the brainstem. Furthermore, symptoms of respiratory failure and postural tachycardia syndrome are likely due to dysregulation of the cardiorespiratory centers of the brainstem (8,13, 123). Moreover, the brainstem has a regulatory role in maintaining processes within other major systems in the body including the GI and the nervous systems (7).

##### III. Highly inflammatory cytokines and hypersympathetic effects:

Increased IL-6 levels are associated with increased sympathetic activity (7, 8, 9, 13). IL-1, IL-6, and TNF-α are known to result in malaise, body temperature changes, and nauseaas they increase vasodilation and vascular permeability. Based on this evidence, it is hypothesized that if chronic immune activation persists post-Covid, it leads to autonomic dysregulation. Autonomic dysregulation contributes to prolonged autonomic symptoms of volume dysregulation, hypersympathetic and adrenergic states, along with cardiovascular deconditioning with orthostatic symptoms. At the same time, contrary to the vagus nerve and parasympathetic signaling, sympathetic signaling increases production of proinflammatory cytokines such as IL-6 which exacerbates immune and autonomic dysregulation (124). Currently, a clinical trial is looking at the effect of vagus nerve stimulation in long Covid (NCT05630040).

###### Treatment:

In patients with orthostatic symptoms, increased salt and fluid intake early in treatment can improve symptoms via volume expansion. Furthermore, sleeping in the head-up position while wearing abdominal binders or compression garments can reduce venous pooling and consequent symptoms of orthostatic insufficiency. Therefore, such treatments can be considered in long Covid autonomic syndrome. From a pharmacological stand point, several blood volume expanders (including desmopressin, fludrocortisone, and erythropoietin), heart rate inhibitors (such as propranolol, pyridostigmine, and ivabradine) in orthostatic syndrome with postural tachycardia, vasoconstrictors (examples are midodrine, droxidopa, methylphenidate, and octreotide), Sympatholytic drugs (including clonidine, and methyldopa), and other medications, such as modafinil have been previously shown to help with such autonomic responses (125). However, in the complex picture of long Covid the safety and efficacy of these medications should be considered and further investigated. Currently, a clinical trial is looking at the effect of ivabradine in long Covid Postural Tachycardia Syndrome (NCT05481177).

Another case series study found improvement in patient autonomic symptoms when the cervical sympathetic chain activity was blocked with local anesthesia. They argued that post-Covid, the autonomic nervous system needs to “reboot” in order to recover from damages caused by infection (126). A current clinical trial is looking at the effect of the sympathetic block on long Covid (NCT05638620).

### G. Dermatologic sequelae

In the studies of long Covid-19 patient dermatological complaints, only 3% were found to have skin rash symptoms after 6 months. However, 20% of patients had symptoms of hair loss at 6 months (105). One study noted some individuals developed a skin condition called perniosis, in which skin becomes erythrocyanotic in cold temperature (127).

#### Mechanisms of dermatologic abnormalities

##### I. Microvascular and endothelial dysfunction:

During acute Covid-19 infection damage to the microvascular and endothelial structure along with hypoxia and inflammation leads to tissue damage in many organs including including lungs, brain, heart, kidney, and skin (96, 128, 129). These changes can cause necrosis of the tissue. Specifically in the skin, such changes lead to endothelial swelling, thrombosis and fibrinoid necrosis of surrounding tissue. These features known as “Covid toes” have been found in the toe skin of Covid-19 and long Covid patients (129).

###### Treatment:

For COVID-19-related urticaria, Shanshal suggested use of low-dose systemic steroids to manage underlying hyperactivity of the immune system (130). Topical steroids were suggested for management of maculopapular and erythematous rash of Covid-19. In cases of pernosis, current guidelines instruct patients to use warm clothing and gloves to keep hands warm and to stop smoking. Topical corticosteroids and nifedipine are also other commonly prescribed medications.

### H. Immunologic sequelae

Two diseases related to immunologic manifestation of long Covid are Guillain-Barré syndrome and Multisystem inflammatory syndrome in children.

#### Mechanism of Guillain-Barré syndrome

Guillain-Barré syndrome (GBS) is an inflammatory disorder that occurs in the host after infections by way of molecular mimicry and autoimmune dysregulation that damage peripheral nerves and nerve roots. The most important diagnostic hallmark of GBS is CSF albuminocytologic dissociation, whereby there is an increase in CSF protein without elevation of white blood cells. GBS has been previously reported to occur following Covid-19 infection (131). At the time, the Covid-19-associated GBS was found to share similar characteristics as classic post-infectious GBS with similar pathogenic mechanisms. Furthermore, it was postulated that the cytokine storm caused by Covid-19 contributes to the worsening of the immune dysregulation process that leads to GBS. However in cases of long Covid GBS, delayed GBS manifestation without acute vascular change or para-infectious responsehave been noted. It is hpothosized that this may simply be due to immunomodulatory medications such as dexamethasone treatment of acute Covid-19 respiratory symptoms (132). Nonetheless, we speculate lingering autoantibody productions and ongoing autoimmune responses can explain the GBS of long Covid among many other autoimmune diseases. Again, further investigation is required to determine the exact mechanisms of such phenomena.

##### Treatment:

A systemic review has shown that intravenous immunotherapy (IVIG, 2 g/kg divided over 5 days) is the most frequently used treatment for Guillain-Barré symptoms of long Covid. However, immunotherapy did not achieve favorable outcome in more than 25% of patients (133). More studies are needed to determine other treatment options for individuals who fail the IVIG regimen.

#### Mechanism of multisystem inflammatory syndrome in children (MIS-C)

MIS-C is one Covid-19 sequelae in children and young adults that can lead to serious long-term effects. MIS-C was first reported in a pCovid-19 positive 6-month-old infant who had symptoms similar to Kawasaki disease (KD). The patient did not exhibit cough but had fever and developed erythematous polymorphous maculopapular rashes, peripheral edema, pain, and conjunctival injection with left-shifted white blood cell count, elevated C-reactive protein (CRP) and erythrocyte sedimentation rate (134). MIS-C has mostly been reported in post-Covid individuals after a negative PCR but with positive antibody (135). Previously, an association between coronavirus (SARS-CoV) and Kawasaki disease was reported as well (136). MIS-C cases can present similarly to toxic shock, KD, or a hybrid of both diseases (135, 137).

Some of the distinguishing factors of Kawasaki and MIS-C disease associated with long-Covid includes age of (onset which was older in long Covid), leukopenia with marked lymphopenia, thrombocytopenia, and other signs of long Covid sequelae such as GI, CSF and myocarditis symptoms (138, 139). While initial manifestation of KD is typically in children younger than 5 years old, MIS-C has been reported in individuals as old as 19 (140). The disease also involvesthe cardiovascular system which suggests that immune responses have a major role (141). Symptomatic myocarditis with cardiogenic shock is one of the “atypical” Kawasaki symptoms in this disease (140). Currently clinical studies are following the long-term effect of MIS-C.

Another unique feature of MIS-C is the manifestation of coronary artery aneurysm in some patients (138). The pathophysiology of the disease is still unclear;however, some studies have suggested autoimmune and viral mimicry of the host to be involvedand serological studies have shown elevated cytokine IL-6, IL-8 and TNF-α (142). Currently, clinical trials are further looking at long Covidin the pediatric population (NCT05566392, NCT05582512).

##### Treatment:

Intravenous immunoglobulins, anticoagulation and low-dose aspirin, and immunomodulatory therapy for KD are used to control MIS-C (138, 140, 143). Furthermore, serial echocardiogram and electrocardiogram are recommened for patients with cardiovascular manifestations as is treatment with vasoactive agents and inotropes (144). The efficacy of these treatments is currently being investigated. It is also recommended that similar to KD these patients should be followed by a cardiologist and receive regular echocardiography for possible cardiovascular and vasculitis sequelae (134, 140).

##### I. Gastrointestinal sequelae

Among the GI symptoms of long Covid are heartburn and acid reflux, nausea, diarrhea, loss of appetite abnormal liver enzymes (145) as well as liver injury, increased AST and ALT (146). The coronavirus family has been shown to readily infect the gastrointestinal (GI) tract (7). In some cases, GI symptoms manifest before any other symptoms of Covid-19. GI symptoms of initial Covid-19 include nausea, vomiting, diarrhea, abdominal pain and reflux (147). Many current investigators report that viral shedding in faeces persists for nearly 5 weeks even after Covid-19 respiratory sample real-time RT-PCR tested negative consecutively for SARS-CoV-2 RNA (148). This is not surprising as it has long been known that SARS-CoV viral RNA can be detected in stool specimens more than 10 weeks post infection (149). Such data suggest that both SARS-CoV and SARS-CoV-2 (Covid-19) can have fecal-oral transmission for a prolonged period of time even in the absence of respiratory symptoms and/or negative respiratory PCR tests. These data suggests that Covid-19 can produce long term effects in GI tract physiology and anatomy even absent respiratory tract infection.

#### Mechanism of gastroenteric symptoms

##### I. Direct invasion of enterocytes and hepatocytes:

The mechanism of gastrointestinal tract and liver infection of SARS-CoV and Covid-19 entry is hypothesized to be through ACE2 receptors (7, 8, 9).

##### II. Inflammation, immune abnormality and impaired gut-brain interaction:

**Figure 6:**
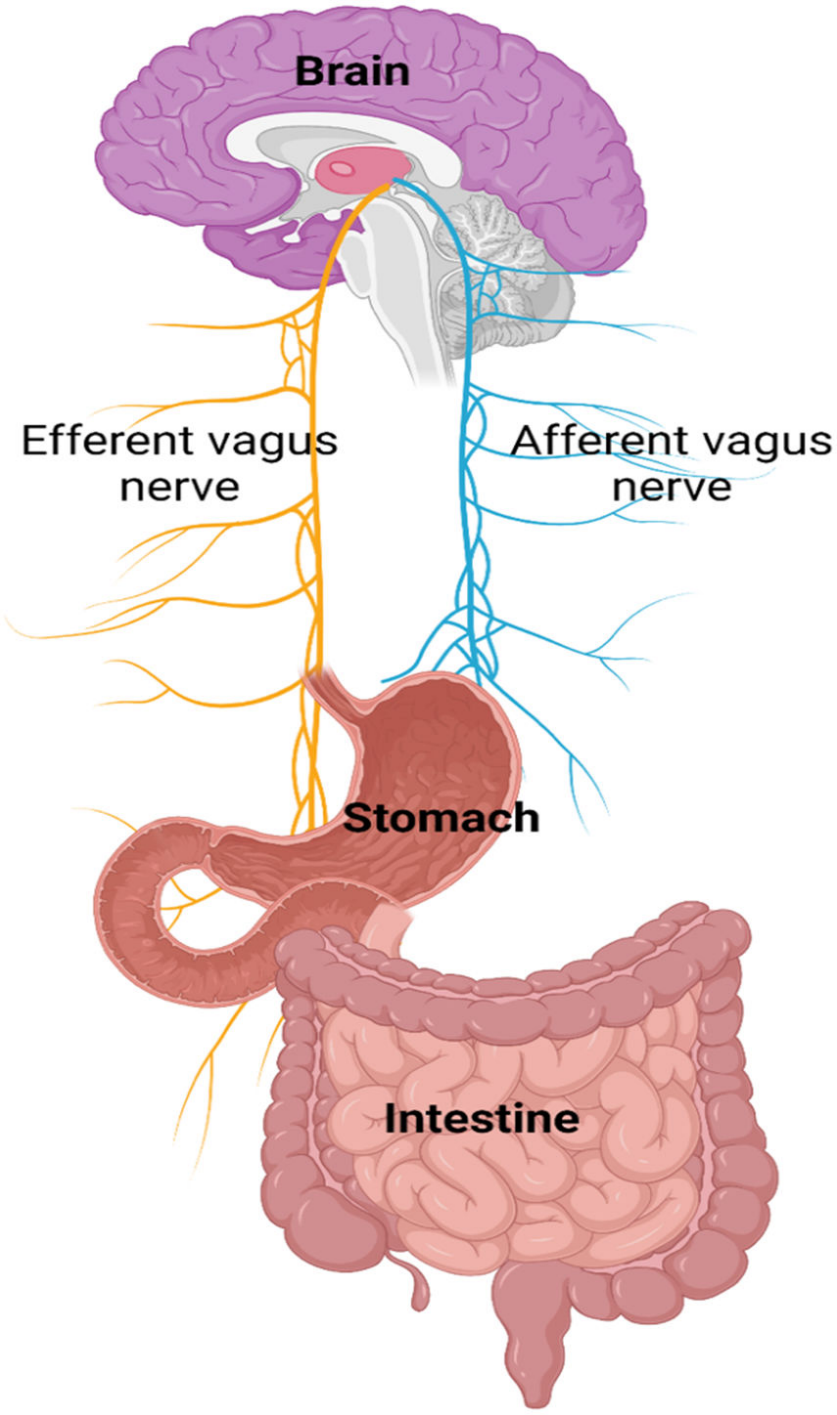
Efferent and afferent vagal nerve are critical in gut-brain interaction.

Long Covid GI symptoms and associated inflammation have processes similar to functional gut-brain interactions common in gastrointestinal disorders linked to increased gut permeability, dysbiosis, bile malabsorption, gut dysmotility and changes in gut endocrine physiology (7, 9, 150). Both afferent and efferent vagal nerves play a ceitical role in the gut-brain interaction (Figure 6). While the mechanisms of long Covid GI manifestations remain under investigation, one focus is on the role of cytokines. At 90 days post-Covid-19 infection, concentrations of CRP and Alanine aminotransferase (ALT) are higher in patients with gastrointestinal sequelae compared to those without (145). The role of cytokines in GI tract activity ranges from regulation of contractility and motility to maintenance of integrity and function (151). Increased IL-1, IL-6, and TNF-α is associated with irritable bowel syndrome (152). Furthermore, there is evidence of increased GI permeability and loss of GI integrity in post-infectious irritable bowel syndrome (153). Therefore, it is plausible that gastrointestinal symptoms such as diarrhea seen in long Covid may be due to possible elevations of IL-1, IL-6, and TNF-α production causing post-infectious long term loss of gut integrity. Moreover, one study of GI-related long Covid without recent CMV infection, reported that the immune system exhibited an abnormal response of involving bystander CMV-specific T cells along with Covid-19 specific CD8+ T cells (7, 154).

##### III. Dysbiosis:

Microbiota are altered in patients with long Covid (7). Such distortions in the gut microbiome can lead to long Covid. Furthermore in severe acute Covid-19, disruption of gut barrier integrity and increased tight junction permeability leads to the translocation of gut microbiome into the blood stream (7). Additionally, several species of viruses, bacteria, and fungi lost during acute Covid-19 infection are known potential gut immune modulators (155, 156). These studies postulate thate loss of natural gut virome, bacteriome, mycobiome along with impaired immunity and increased inflammatory responses of the gut during acute Covid-19 infection can lead to the growth of opportunistic microorganisms, causing dysbiosis and the emergence of long Covid (157). One of the species lost in Covid-19 associated dysbiosis is *Faecalibacterium prausnitzii* which has been shown to have a role in the inhibition of production of pro-inflammatory cytokines such as IL-1, IL-6, and TNF-α (7, 158). Loss of *F. prausnitzii* from the microbiota further increases inflammatory cytokines associated with Covid-19 symptomatology and, therefore, can result in long-term signs of irritable bowel disease and gut inflammation. The loss of such valuable bacteria from the gut allows opportunistic gut pathogens to proliferate which can further exacerbate long Covid GI symptoms (158). Currently a clinical trial is looking at the significance of gut permeability in long Covid (NCT05612087). Future studies should also investigate the role of regulating gut microbiota for faster recovery from long Covid.

##### IV. Viral persistence in the GI tract:

Viral shedding in feces persists in some Covid-19 survivors long after recovery from acute Covid-19 (159). Thus, Covid-19 may continue to infect and affect other organs (160, 161).

### J. Hepatic sequelae

#### I. Direct invasion:

The mechanism of hepatic impairment in long Covid is poorly understood. While hepatocytes express lower levels of ACE2 compared to enterocytes, they also express a protein called high-density lipoprotein scavenger receptor B type 1 which can facilitate ACE-2 dependent entry of Covid-19 into hepatocytes (162). Therefore, hepatocytes are directly infected by Covid-19 (162).

#### II. Gut-liver axis and exposure to inflammatory microflora:

As noted earlier, viral shedding in the feces of Covid-19 survivors render the post-Covid gut a highly inflammatory environment (159). Altered intestinal permeability exposes the liver to luminal microflora containing IL-1β, IL-6, and TNF-α which exacerbates inflammatory responses in the liver and causes further hepatic injuries. It is also hypothesized that liver exposure to cytokines as well as oxidative stress and changes in fatty acid metabolism during Covid-19 leads to downstream metabolic hepatocyte stress with lipid accumulation and formation of non-alcoholic fatty liver, or its newer term, metabolic associated fatty liver disease (163).

##### Treatment:

Enrichment of gut microbiome, through transplant, probiotics and/or microbiota-derived metabolites, and engineered symbiotic bacteria are some of the therapeutic avenues to combat GI symptoms of long Covid (164). These pathways can also benefit the gut-liver axis and help repair liver tissue in long Covid. Currently, a clinical trial is looking at some of these potential treatments for GI-related long Covid (NCT05080244).

### K. Pancreatic sequelae

Pancreatic injury, pancreatitis have been shown to be associated with long Covid (146).

#### Mechanism of pancreatic impairment and diabetes

##### I. Direct invasion and inflammation:

ACE2 protein is expressed in pancreatic β cells, adipose tissue, intestinal cells, and renal cells (8, 13). Therefore, Covid-19 infection can have a collective effect on glucose metabolism and can contribute to new onset of diabetes or worsen preexisting diabetes. Currently, research at CoviDIAB Project is collecting data on the relationship between Covid-19 and diabetes. As is explained in the “Viral antigen persistence (Persistent viremia)” section, chronic exposure to superantigens due to inefficient immune response and incomplete immunity can lead to systemic inflammation. Inflammation caused by Covid-19 in pancreatic β cells leads to their apoptosis which induces development of diabetes in long Covid (7, 146).

###### Treatment:

No specific treatment other than usual care and diabetic medications have been proposed for pancreatic impairment due to long Covid. Future efforts should look at early diagnosis and protective interventions to reverse impairments sustained or newly arisen after acute Covid-19 diagnosis.

### L. Splenic sequelae

A cross-sectional study found mild impairment in spleen function of 6% of Covid-19 patients after more than 3 months post-recovery (165). Reduction in T and B lymphocyte, and atrophy of splenic lymphoid follicles in some long Covid patients have been reported (9, 146).

#### Mechanism of splenic dysfunction

##### I. Direct invasion of the spleen:

ACE2 receptors are expressed in the spleenand, therefore, splenic tissue may be subjected to damage through viral direct attack (165). Chronic systemic inflammation existing long after acute Covid-19 infection can also excaerbate this process.

###### Treatment:

No specific treatment has been proposed for splenic dysfunction of long Covid not due to thrombotic tissue damage of severe acute Covid-19. Some have logically proposed anticoagulation treatment of long Covid cases that entail splenic thrombosis.

### M. Renal sequelae

#### Mechanism of renal impairments

##### I. Vasculitis and endothelial injuries:

It has been suggested that post-Covid renal impairment is linked to activation of coagulation factors as well as endothelial injuries, as will be discussed later in this review (72, 105, 128). Changes in RAAS, emergence of vasculitis and endothelial dysfunction are also speculated to be other possible mechanisms caused by Covid-19 (8, 128). Direct invasion of kidney’s podocytes and ACE2 expressing cells of the proximal tubule is one potential mechanism of kidney damage in long covid (166).

###### Treatment:

No specific treatment has been proposed for renal impairment of long Covid. Future studies must further explore the prophylaxis and treatment options for kidney disease of long Covid.

### N. Thyroid sequelae

#### Mechanism of thyroid dysfunction

##### I. Inflammation and interferon treatments:

A recent study showed that transient thyroid function abnormality may be found at 3 months post-Covid-19, but usually resolves by month 6. They found Covid-19 infection did not cause change in thyroid autoimmunity. However, interferon treatment increased anti-thyroid antibody titers (167).

###### Treatment:

Current treatment closely follows standard of care for each type of thyroid disease manifestation, hypothyroidism, hyperthyroidism, or subacute thyroiditis, e.g. propranolol for treatment of palpitations caused by hyperthyroid states and other specific symptomatic treatment (168). Future studies must investigate preventative and therapeutic measures for thyroid dysfunction of long Covid and whether limiting or optimizing interferon treatment may improve outcomes.

### O. Obstetrical and gynecological sequelae

A recent study has shown symptoms of long Covid in pregnant women included fatigue, hair loss and lack of concentration, though this was not statistically different from the general population (169). Covid-19 affects the duration, frequency, regularity, and severity of menstrual pain (170). It is important to consider the short-term and long-term effect of Covid-19 on women’s health and fertility.

#### Mechanisms of menstrual cycle changes

The menstrual cycle is controlled through feedback from multiple organs including the hypothalamus, pituitary, ovaries, uterus, prostaglandins, and neuroendocrine factors. It is postulated that changes to any of these organs during long Covid may potentially disrupt menstrual cycle. Likewise, Covid-19 may directly attack neurons of CNS such as the hypothalamus. Furthermore, cytokine changes and inflammatory responses during acute and long Covid exacerbates damages to every organ involved and can therefore lead to menstrual dysregulation and increased severity of pain during menses (171).

##### Treatment:

While hormonal therapy is plausibly one of the treatment options for changes in menstruation due to long Covid, further studies are required to determine the underlying cause of such changes and enhance therapeutic avenues.

#### III. Potential Contributors to long Covid-19 Disease

**Figure 7:**
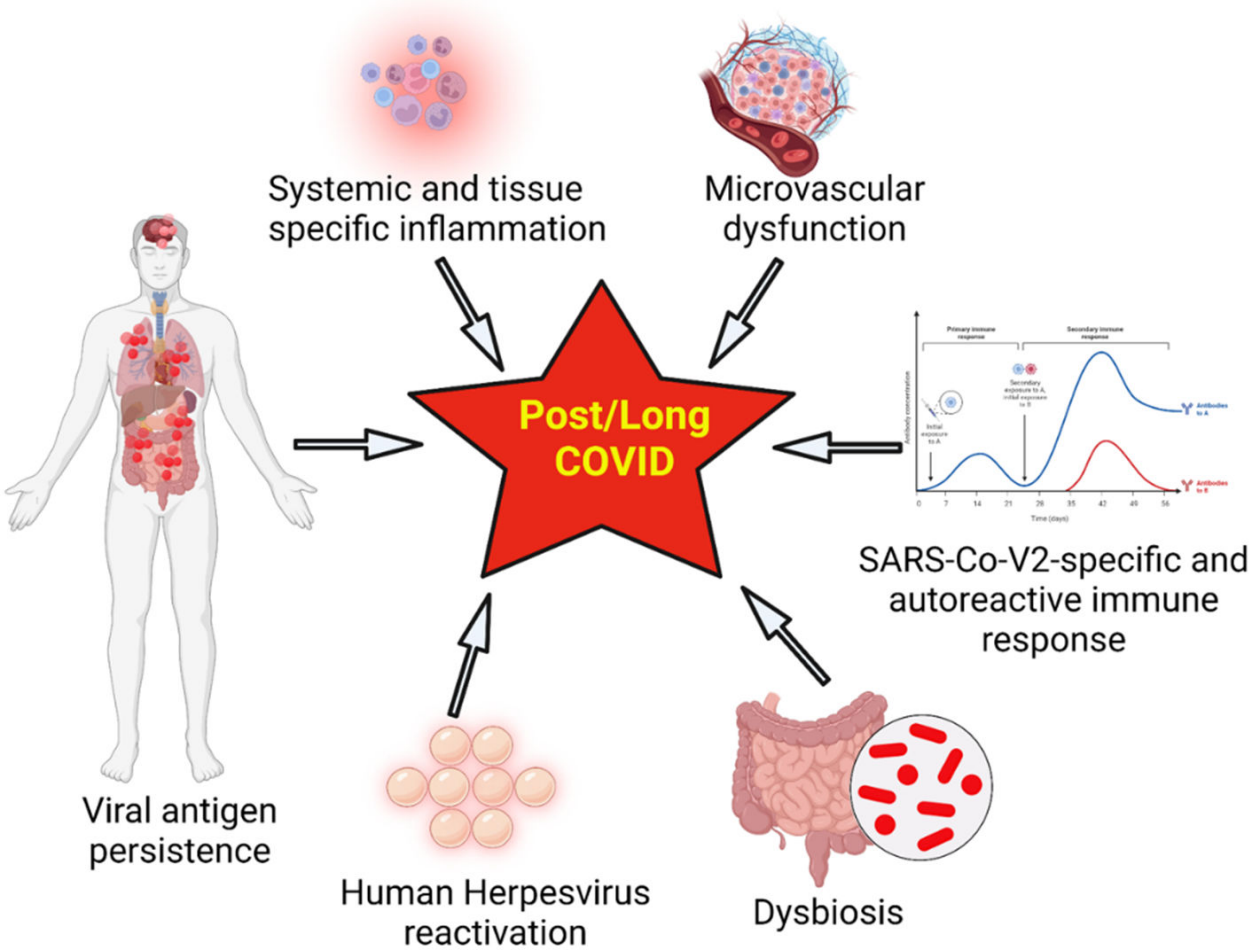
Key factors involved in the underlying pathophysiology of post/long Covid.

There are several key factors that are responsible for the underlying pathophysiology of long Covid. The pathophysiological features are primarily due to cytokine storm. The critical factors include systemic and tissue specific inflammation, microvascular dysfunction, activation of coagulation factors, persistence of viral antigen, reactivation of human herpes virus, SARS-Co-V2 specific and autoreactive immune response, and dysbiosis (Figure 7). Each of these factors are discussed below.

##### 1. Systemic and tissue-specific inflammation

###### Mast Cell Activation Syndrome:

There is emerging evidence suggesting a potential link between mast cell activation syndrome (MCAS) and long Covid. Mast cells are known to regulate functions of many immune cells in a wide variety of bodily tissues (172). It has been shown that mast cells express ACE2 and overactivation of mast cells leads to myriad signs and symptoms within the overarching syndrome called Mast Cell Activation Syndrome. Much as in long Covid, MCAS symptoms manifest in many systems including digestive track, dermis, cardiovascular and nervous system (173).

Studies that link MCAS to Covid-19 have found that symptoms of brain fog in long Covid are similar to chemofog for cancer patients as well as chronic fatigue syndrome and MCAS. All these diseases have some component of brain inflammation. It is postulated that in acute Covid-19 activation of mast cells can lead to recruitment of immune cells and release of IL-1, IL-6, and TNF-α (174). Several MCAS biomarkers including histamine, prostaglandin D2, serum tryptase are also elevated in long Covid (175). While the exact causes of long Covid are yet to be fully understood, we hypothesize that the invasion of mast cells due to Covid-19 infection leads to MCAS-like processes in a range of tissues. Overactivated mast cells result in persistent inflammation, immune dysregulation and potentially lead to sustained systemic inflammation in various organs in the body.

####### Treatment:

Luteolin and quercetin, natural flavonoids found in various plant-based foods, such as parsley, inhibit viral entry into host cells and inhibit inflammation (176). Quercetin and luteolin can potentially be used to develop a new class of RNA-dependent RNA polymerase inhibitors for treatment of Covid-19 (177). In another study on a Covid-19 pseudovirus system, inhibition of Covid-19 was achieved upon a dose-dependent administration of luteolin and quercetin (178). There are several molecular studies that shown flavonoids may potentially regulate inflammation induced long Covid (179). Leuteolin also inhibits mast cells and can inhibit TNF-α, IL-1, as well as chemokines CCL2 and CCL5 (81, 180).

CCR5 is one of the important transmembrane proteins involved in viral entry as well as the function of memory T-lymphocytes, macrophages, and immature dendritic cells. An ongoing clinical trial is testing efficacy of monoclonal antibodies such as leronlimab (CCL-5 blocker) to reduce the inflammatory response post-Covid-19 (NCT04343651) (181). Other clinical trials are investigating the role of tocilizumab, an IL-6 receptor blocker, in long-covid (NCT04330638).

##### 2. Coagulation activation

Several mechanisms of Covid-19-associated coagulopathy have been elucidated, with respect to thromboinflammatory and hypercoagulable states (8, 72). In the following section we explain two of the major mechanisms related to coagulation activation (complement cascade activation, microvascular and endothelial injury, and platelet activation).

###### Treatment:

Anticoagluation therapy for prophylaxis or/and treatment of long Covid should be investigated.Indeed, in 2020 the American Thoracic Society/European Respiratory Society guideline recommends 3 months of treatment of venous thromboembolic events in severe Covid-19 (182).

###### 2A. Complement cascade activation:

Several complement proteins and tissue factor, platelet, neutrophil extracellular traps (NET)s were found to be increased in plasma of severe acute respiratory syndrome coronavirus and Covid-19 patients (9, 183, 184, 185). The persistence of these factors in the tissue can contribute to symptoms of long Covid.

####### Treatment:

Small studies have shown that use of complement inhibitors during acute Covid-19 can reduce Covid-19-associated complement activation (186). Such treatments can also be considered in the hypercoagulable states associated with long Covid.

###### 2B. Microvascular and endothelial injury, and platelet activation:

Severe endothelial damage in the vasculature of lung, heart, kidney, liver, and intestine of Covid-19 patients (8, 96, 128, 129). Such findings causally suggest disruption of ACE2 receptor downstream cascade in these vascular beds. These impaired microcirculatory findings are collectively known as Covid-19-endothelitis (8, 96, 128, 187). It is important to note that capillary and endothelial damage caused in these tissues during Covid-19 not only contributes to microthrombosis but also can disrupt blood and tissue oxygenation, subsequently leading to necrosis and impairment of tissue function (8, 72, 96, 129). Further evidence of microvascular and endothelial damage hypothesis was substantiated through studies that report von Willebrand Factor (vWF) elevation in the blood of severe Covid-19 patient, as is consistent with endothelial injury and dislocation of this protein into plasma (9, 188). Consequently, activation of vWF allows for platelet activation and aggregation (189).

####### Treatment:

Use of antioxidant and cholesterol-lowering therapies, ACE inhibitors, and anti-TNF-α during acute phase of Covid-19 has been proposed asemans to stabilize endothelium (9, 190). Such considerations are especially important in comorbidities that can lead to endothelial dysfunction; i.e., smoking, hypertension, diabetes, obesity, male sex, and history of cardiovascular diseases (128).

##### 3. SARS-COV-2 specific and autoreactive immune responses

Covid-19 can evolve into long Covid through long-term activation of host immune response (191). Some autoantibodies are involved in a variety of immunological processes, from lymphocyte activation to leukocyte recruitment and traffickingand can directly target vascular, CNS, and connective tissues. Other studies have shown Covid-19 leads to persistent autoimmune activation and proinflammatory states months after resolution of the acute phase. This is though to be mediated with upregulation TNFα, IL-6, IL-1β, IL-13, granulocyte colony-stimulating factor (G-CSF), interleukin (IL)-17A and many other inflammatory markers (192).

Covid-19 also has been shown to drive production of antibodies that cross-react with self-antigens found in various tissues in the body such as the brain, heart, lung, gut, and kidney (193). It has been shown that there is a link between long Covid and Guillain-Barré syndrome. As was previously discussed in MIS-C section, Kawasaki-like disease in children and young adults is another manifestation of autoimmune responses in both acute Covid-19 as well as long Covid (141).

In early studies of Covid-19 infection it was speculated the virus may generate autoantibodies against ACE2 or its complexes which can potentially lead to delayed complications of Covid-19 (194). Later studies found autoantibodies against ACE2 post-covid infection decrease activity of ACE2 leading to increased Angiotensin II concentration. which further exacerbates the proinflammatory state seen in symptoms of long Covid (195, 196). Some studies have shown presence of antiphospholipid antibodies in patients with Covid-19 can lead to self-immune attacks, vascular inflammation, and emergence of coagulopathies (115). Such can also lead to NETosis and persistent inflammatory processes in large vessels which affects long-term cardiovascular health (116). Another study has shown production of autoantibodies against nociception receptor, β2-adrenoceptor, α1-adrenoceptor, endothelin receptor, muscarinic receptor, angiotensin II AT1 receptor, and MAS receptor in post-Covid patients all of which affect both the neurological and cardiovascular systems. Moreover, another study found association between chronic increases ofanti-nuclear/extractable-nuclear antibodies (ANAs/ENAs) and persistence of Covid-19 symptoms contribute to long Covid (117, 197).

###### Treatment:

One treatment option for autoimmune dysregulation in post-Covid syndrome is the use of immunoadsorption to effectively remove autoantibodies (198). Clinical trials are looking at the effect of immunoadsorption in reduction of symptoms of post-Covid-19 chronic fatigue syndrome (NCT05710770, NCT05629988). Otherimmunomodulatory medications used for treatment of autoimmune diseases have promising results in long Covid (199, 200). As also discussed in the MIS-C section, the role of tocilizumab as one of the immunomodulator is being investigated both in KD as well as in long Covid (NCT04330638). Another clinical trial is investigating the role of autoimmunity development in long Covid syndrome (NCT05459506).

##### 4. Viral antigen persistence (persistent viremia)

Certain genetic variations in humans cause defects in innate immunity against viruses, e.g., as a mutation in toll-like receptors and mannose-binding lectin. In patients with such defects, viral antigens can persist in tissues and confer higher risk of long Covid (201).

Studies on viral shedding in feces have shown that some cases of Covid-19 infection persist long after recovery from major symptoms (159). Therefore, Covid-19 has the potential to continue infecting various organs (161). Persistence of Covid-19 can also be due to incomplete immunity (202). Severity of an immune response is not a determinant of effective immunity as in severe ineffective immunity with superantigens. Superantigens can result in strong but nonspecific and incomplete immunity in respiratory viruses (9). Furthermore, chronic superantigen exposure can lead to long-term systemic inflammation which explains many systemic inflammatory symptoms related to long Covid, including the development of diabetes long after disease recovery.

##### 5. Reactivation of latent viruses

Covid-19 results in reactivation of several dormant herpes virus in human such as Epstein–Barr virus (EBV, herpesvirus 4), cytomegalovirus (herpesvirus 5), Roseola (herpesvirus 6) (203). This might be caused by decreased immunity due to Covid-19 infection with consequent reactivation of chronic or dormant infections. Furthermore, it has been found that EBV lytic replication leads to increased ACE2 expression on epithelial cells which facilitate Covid-19 entry into cells (204). Therefore, it can be postulated that individuals with latent EBV are more prone to Covid-19 infection and one negative impact of Covid-19 infection reactivation of EBV contributing to signs of long Covid. Moreover, multiple infections lead to stress, mitochondrial fragmentation, and impaired metabolism — changes that may contribute to symptoms of fatigue and the persistence of complex symptoms in long Covid.

#### III. Risk factors for long Covid

Studies have shown severity of acute Covid and history of hospitalization are major risk factors for persistence of symptoms as well as development of long Covid (205). Furthermore in hospitalized patients, older age and female sex have been shown to increase risk of long Covid (205). Sex differences of risk are hypothesized to bedue to higher ACE2 expression in females and greater unfavorable psychological factors including stress, anxiety, depressive disorder, and sleep disturbances in females than males (206). These risk factors do not seem to impact symptoms of long Covid in non-hospitalized patients (207). Manifestation of more than 5 symptoms during acute Covid-19 is also associated with increased risk of long Covid (205). The presence of other comorbidities has also been shown to increase risk of long Covid.

#### IV. Effects of Vaccines on long Covid

A study carried out by Arnold *et al.* showed that patients who received either an mRNA or adenoviral vector vaccine (Pfizer-BioNTech (BNT162b2) or Oxford-AstraZeneca (ChAdOx1 nCoV-19)) between January to February 2021 did not have worsening of long Covid symptoms, mental wellbeing, or quality of life (208). One other study showed that vaccination in long Covid resulted in worsening of long Covid symptoms (209). Amid this controversial debate, a scoping review study was carried out which determined most studies of patients vaccinated for Covid-19 reported improvement in symptoms of long Covid (210). Some studies showed significant improvement in symptoms of long Covid even after administration of the first vaccine dose (211). Covid-19 vaccination also reduced manifestations of major symptoms of long Covid (212). A clinical trial is currently studying the effect of Covid-19 vaccination status on long Covid (NCT05587868). Another clinical trial is looking at long Covid and vaccination in patients with chronic liver disease as well sd post-transplant individuals (NCT05107271).

One current issue with vaccine efficacy against Covid-19 and consequent long Covid relates to individuals with kidney disease. Previous studies have shown that vaccination against viruses such as influenza and hepatitis B has low efficacy in patients with severe kidney disease due to immunosuppression caused by the uremic waste products (213). Therefore, currently a clinical trial is looking at the long-term efficacy of Covid-19 vaccination in patients with CKD (NCT04841785).

One study has shown persistent short nighttime sleep duration is associated with a greater post-Covid risk in fully mRNA-vaccinated individuals (214). Individuals with short nighttime sleep (6 h) have weaker immune systems and are more likely to develop long Covid syndrome, even after complete vaccination.

Interestingly, early in 2022, rare cases of Covid-19 vaccine related long Covid-like symptoms were discovered. These individuals mostly had symptoms of headache and brain fog. It is hypothesized that such signs may be due to formation of postvaccination autoantibodies (209, 215). Nonetheless, recent studies have shown that not receiving Covid-19 vaccines is associated with higher risk of long Covid (209).

#### V. Future direction in understanding, preventing, and treating long Covid complications

Clearly, long Covid leads to a broad spectrum of symptoms as it affects multiple organs through a wide range of mechanisms. While many treatment options have been proposed and are being investigated, further research is required to enhance our knowledge increase therapeutic avenues. Furthermore, there remain many controversies regarding the impact of vaccination and biological factors on long Covid. We have reviewed how a major part of the complex picture of long Covid can be explained by teasing out and further streamlining roles of multiple factors in diagnosing, and managing the severity of Covid-19 and long Covid.

Nonetheless, more studies of long Covid are needed, particularly to enhance prevention, promote recovery, optimize quality of life, and reduce future patient morbidity and mortality. Moreover, it is important to further investigate the role of long Covid in women’s health, pregnancy and the impact on future generations.
